# Electrochemical Oxidation Induced Multi-Level Memory in Carbon-Based Resistive Switching Devices

**DOI:** 10.1038/s41598-018-38249-0

**Published:** 2019-02-07

**Authors:** Paola Russo, Ming Xiao, Norman Y. Zhou

**Affiliations:** 1 Waterloo Institute for Nanotechnology, University of Waterloo, 200 University Avenue West Waterloo, Ontario, N2L 3G1 Canada; 2 Centre for Advanced Materials Joining, University of Waterloo, 200 University Avenue West Waterloo, Ontario, N2L 3G1 Canada; 3 Multi-Scale Additive Manufacturing Lab, University of Waterloo, 200 University Avenue West Waterloo, Ontario, N2L 3G1 Canada; 4 Department of Mechanical and Mechatronics Engineering, University of Waterloo, 200 University Avenue West Waterloo, Ontario, N2L 3G1 Canada

## Abstract

In this work, we report for the first time the electrochemical oxidation as a technique to improve the electrical performances of carbon-based resistive switching devices. The devices obtained through the anodic oxidation of carbon-structures possess superior electrical performances i.e. a 3-level memory behavior and an ON/OFF ratio two order of magnitude higher than the non-oxidized carbon-based devices. It is demonstrated that the chemical composition of the carbon structures (i.e. percentage of oxygen groups, sp^2^ and sp^3^ carbon atoms) plays a key role in the improvement of the carbon-based devices. The electrochemical oxidation allows the possibility to control the oxidation degree, and therefore, to tailor the devices electrical performances. We demonstrated that the resistive switching behavior in the electrochemically oxidized devices is originated from the formation of conductive filament paths, which are built from the oxygen vacancies and structural defects of the anodic oxidized carbon materials. The novelty of this work relies on the anodic oxidation as a time- and cost-effective technique that can be employed for the engineering and improvement of the electrical performances of next generation carbon-based resistive switching devices.

## Introduction

Carbon materials, such as graphene oxide (GO)^1,2^, reduced graphene oxide (rGO)^3,4^, and carbon nanotubes (CNTs)^5,6^ are attracting growing interest in nanoelectronics as alternatives to traditional silicon-based electronics due to their unique electrical characteristics, optical transparency and flexibility^7–10^. Carbon materials for their high operation speed and scalability^1,11–13^ have been investigated as insulator/semiconductor layer for the fabrication of resistive switching memory devices (RRAM). These type of devices consist of an insulator layer sandwiched between two metallic electrodes, where the resistive switching effect between high resistance state (HRS) and low resistance state (LRS) is induced by the application of an electric field^14,15^. The underlying resistive switching mechanism in these devices is under debate and requires more understanding. Some interpretations suggest that the absorption and release of oxygen functional groups are responsible for the switching behavior in GO, with a mechanism similar to the valence change mechanism (VCM)^16^. Another explanation focuses on the diffusion of metallic ions from the electrodes through the GO layer, similar to the electrochemical metallization mechanism (ECM)^4,17^. Other theories attribute the resistive switching effect to the space-charge-limited conduction (SCLC) mechanism controlled by the presence of defects in the materials, *i.e*. oxygen vacancies, which create charge-carrier traps^15,18–20^. The carbon-based RRAM are promising candidates for non-volatile memory applications due to their good retention performance, high on/off current ratio and good reproducibility^1–4,10,15,19–21^. One of the disadvantages that hamper their development on a large scale is their fabrication processes, which involve high temperatures and pressures and the use of chemicals harmful for the environment^9^. Consequently, a simple, fast, cost effective and eco-friendly method for the fabrication of carbon-based electronics is needed in order to enable their broad production. In our previous work^22^ it was found that the electrical properties of the carbon-based devices could be improved if a control over the chemical composition of the carbon material can be performed. Preliminary results suggested that an increase of oxidation might lead to a device with improved electrical performances.

Here, electrochemical oxidation of carbon structures (Cs) is employed as a simple and time-effective approach for the tailoring and improvement of the performances of the carbon-based devices. To our best knowledge, this technique has not been used as a tool to improve the electrical performances of carbon-based RRAM devices. Indeed, it has been widely used as a technique to study the redox processes involved in the resistive switching mechanisms of different types of RRAM^23–25^, and to increase the electrochemical capacitance of carbon materials by the introduction of oxygen functional groups on the surface of materials^26,27^. In this work, we demonstrate that by electrochemical route it is possible to control the chemical composition of the Cs confirming their potential as a resistive switching material. The electrochemically modified material is easier to obtain than the widely used GO^28^, therefore this approach could be used for the large-scale development of carbon-based memory devices and the chemical composition of other carbon materials (i.e. graphene, carbon nanotubes etc.) could be tailored.

## Materials and Methods

### Synthesis of the Cs

The arc discharge in solution^29–38^ has been widely employed for the synthesis of carbon nanomaterials such as carbon nanotubes and polyynes, linear carbon chains with alternating single and triple bonds. In this work the Cs were prepared combining the arc discharge in water with the electrophoretic deposition (EPD), as we recently reported^22^. In summary, the arc discharge was performed by applying a voltage of 30 V and a current of 10 A between two graphite electrodes for 10 minutes for the synthesis of polyynes, which are the building blocks for the fabrication of the Cs by EPD. Indeed, the solution containing the polyynes was deposited on fluorine-doped tin oxide coated glass (FTO) substrate by EPD. Two FTO substrates were submerged in the polyynes solution and connected to a power supply. The deposition of the Cs was performed for 2 hours at a voltage of 30 V and current of 0.01 A. The Cs obtained at the cathode (Cs@FTO) were then let dry at room temperature.

### Electrochemical oxidation of the Carbon structures (Cs)

The oxidation of Cs was achieved by anodic oxidation in a three-electrode quartz cell where the Cs@FTO sample serve as the working electrode, a platinum wire as the counter electrode and the saturated calomel electrode (S.C.E.) as the reference electrode. The Cs@FTO sample was submerged into an aqueous solution of sodium sulfate (Na_2_SO_4_) 1 M and a potential in the range of [0–0.8] V vs. S.C.E. with a scan rate of 20 mV/s. After the oxidation of the samples, the oxidized Cs (OCs@FTO) were rinsed with deionized water and dried at room temperature.

### Device fabrication

The carbon-based RRAM devices were obtained by depositing aluminum electrodes (100 nm in thickness and 1 mm diameter) on top of the Cs@FTO and OCs@FTO by an e-beam evaporation process. During the electrical characterization, the aluminum acts as top electrode and the FTO is grounded.

### Instrumentations

The morphology characterization of the samples were performed by scanning electron microscopy (SEM) using a ZEISS LEO 1550 FE-SEM at an accelerating voltage of 7 kV. For the HRTEM analysis, the samples were scratched onto lacey carbon grids and observed using a JEOL 2010F at the Canadian Centre for Electron Microscopy (Hamilton, Ontario, Canada). The surface chemical composition analysis was performed by X-ray photoelectron spectroscopy (XPS) analysis using a multi-technique ultra-high vacuum imaging XPS microprobe spectrometer (Thermo VG Scientific ESCALab 250) with a monochromatic Al-Ka 1486.6 eV X-ray source. Raman analysis was performed with a 50x objective, and the spectra were acquired with a laser wavelength of 633 nm at a power of 0.1 mW. For the electrochemical oxidation, a Gamry Potentiostat (Series 300) was employed. The electrical measurements of the carbon-based devices were performed with a Keithley 2602 A source meter at ambient conditions.

## Results and Discussion

### Material Characterization

**Figure 1:**
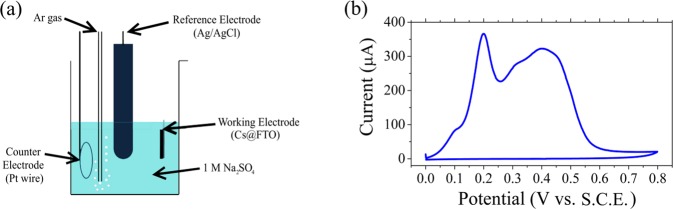
(**a**) Schematic of the three-electrode quartz cell employed for the oxidation of the carbon structures. (**b**) CV of Cs@FTO in 1 M Na_2_SO_4_ aqueous solution. The scan rate is 20 mV/s.

A schematic of the system employed for the anodic oxidation is depicted in Fig. 1(a), while the cyclic voltammogram (CV) curve obtained upon the oxidation of the sample is shown in Fig. 1(b).

The changes in the CV shape of the Cs are due to the chemical modification of their surface induced by the anodic oxidation. To demonstrate this, we carried out the anodic oxidation of a FTO sample without the deposition of the Cs (See Fig. 1S in the Supplementary Data). Noteworthy, no peaks were detected, confirming that the peaks in the [0–0.8 V] range (Fig. 1b) arise as a result of the oxidation of the Cs. From the analysis of the peaks it is possible to assess that the peak at 0.2 V is attributed to the formation of hydroxyl groups (-OH) occurring at carbon defects sites according to reactions 1 and 2, displayed below^39,40^. The broad peak in the range of [0.3–0.5] V is attributed to the formation of carbonyl, carboxyl (HO-C-C=O-), and epoxy (O-C-O) groups at the surface of the Cs^26,40–42^.

1 $${\rm{E}}={\rm{0.207}}\,{\rm{V}}$$

2 $${\rm{E}}={\rm{0.518}}\,{\rm{V}}$$

**Figure 2:**
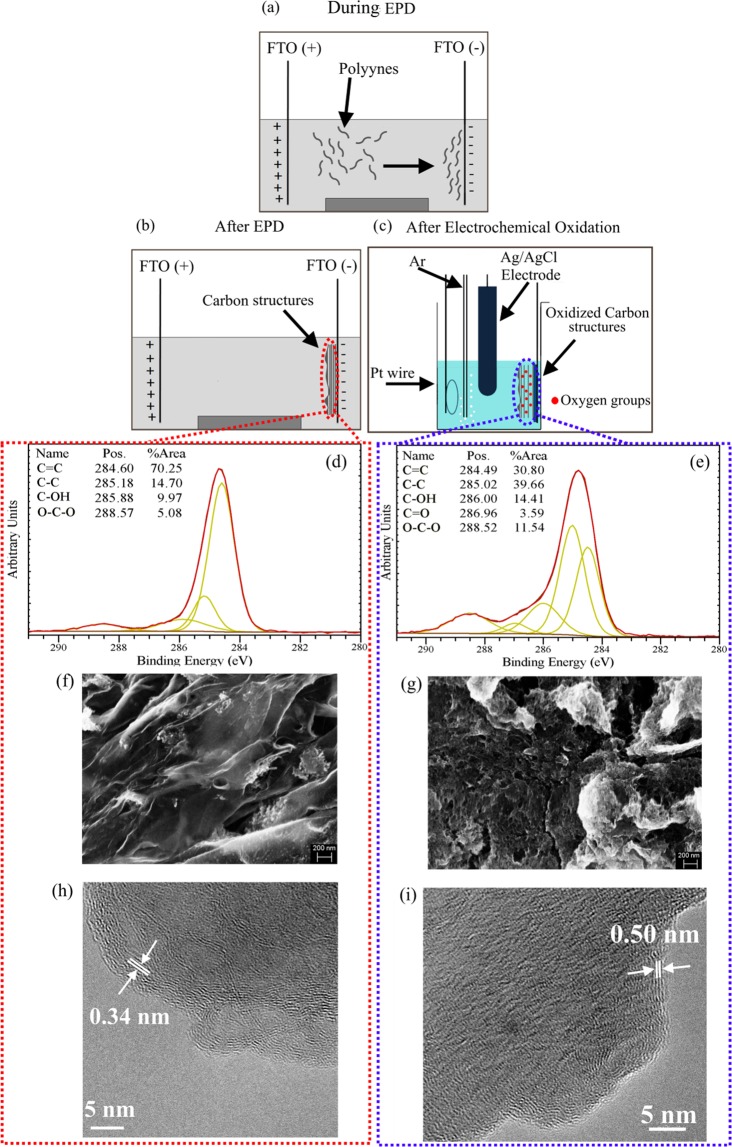
(**a,b**) schematic of the bottom up synthesis of Cs upon deposition of polyynes and (**c**) electrochemical oxidation of the synthesized Cs; (**d,f,h**) C 1 s XPS spectra, SEM and TEM images of the Cs before and (**e,g,i**) after the electrochemical oxidation. In (**h,i**) it is possible to notice that the d-spacing of the as-prepared Cs is 0.34 nm, which increases to 0.5 nm upon oxidation.

Figure 2a,b displays a schematic for the synthesis of Cs upon electrodeposition of the polyynes on the FTO substrate, as demonstrated in our previous work^22^. The deposited Cs@FTO samples were then electrochemically oxidized (Fig. 2c) and characterized by XPS, SEM and TEM (Fig. 2d–i). From the XPS results carried out before the oxidation (Fig. 2d), it is possible to note a mild oxidation of the pristine Cs samples, probably induced by EPD process^22^. The peak at 284.60 eV is attributed to C=C bonds, while the C-C bonds give rise to the peak at ~285 eV.

The as prepared sample contains hydroxyl and epoxy groups and the related peaks are situated at 285.88 eV and 288.57 eV, respectively. The XPS of the OCs is displayed in Fig. 2e and it is possible to observe a new peak at 286.96 eV, which can be attributed to the presence of carbonyl group. Compared to the pristine Cs, in the OCs sample the percentage of sp^2^ carbon atoms attributed to C=C bonds decreases, while the percentages of sp^3^ carbon atoms, hydroxyl groups and epoxy groups increase. These findings are in agreement with the results obtained from the analysis of the CV curve previously discussed, which confirm the oxidation of the Cs through electrochemical route. SEM and TEM analyses were carried out to study the morphology and structure of the Cs and OCs samples. The results are displayed in Fig. 2f,g and Fig. 2h,i, respectively. It was observed that the oxidation of the Cs induced a morphological and structural modification. An increase of the d-spacing from 0.34 nm, typical of graphitic structures, to 0.50 nm occurred, due to the presence of oxygen groups within the Cs layers^43,44^. This is illustrated in Fig. 2c as red dots between the layers. The TEM cross section analysis displayed in Figures S2 and S3 further confirms the oxidation of the electrochemical oxidation of the Cs. In particular, when analyzing the composition maps it was found that the atomic percentage of oxygen and carbon in the Al/Cs@FTO was 11.94% and 88.06%, respectively. Instead, in the Al/OCs@FTO device, the atomic percentage of oxygen and carbon was 63.04% and 36.96%, respectively. The Raman characterization was performed on the Cs and OCs samples and the spectra are displayed in Figure S4. A typical Raman spectrum of carbon materials displays three main characteristic peaks: the D, G and 2D bands. The D band is induced by defects in the crystalline structures and it is related to the size of the in-plane sp^2^ domains^45^. The in-plane vibration mode of sp^2^ carbon atoms gives rise to the G band, while 2 D and D + G bands are originated by the second order Raman vibration modes^46,47^. The I_D_/I_G_ and I_2D_/I_G_ ratio can give information on the reduction degree of graphene oxide as it is inversely proportional to the average size of the sp^2^ domains^45,48–50^. In the OCs sample, the I_D_/I_G_ ratio increased, thus confirming their electrochemical oxidation, which is further supported by the decrease of the I_2D_/I_G_ ratio, from 0.28 to 0.20, indicating a decrease of sp^2^ domains^27^. The characterization analysis carried out on the Cs and OCs samples confirmed the electrochemical route as an efficient and time-effective method for the oxidation of the carbon materials.

### Electrical Measurements

**Figure 3:**
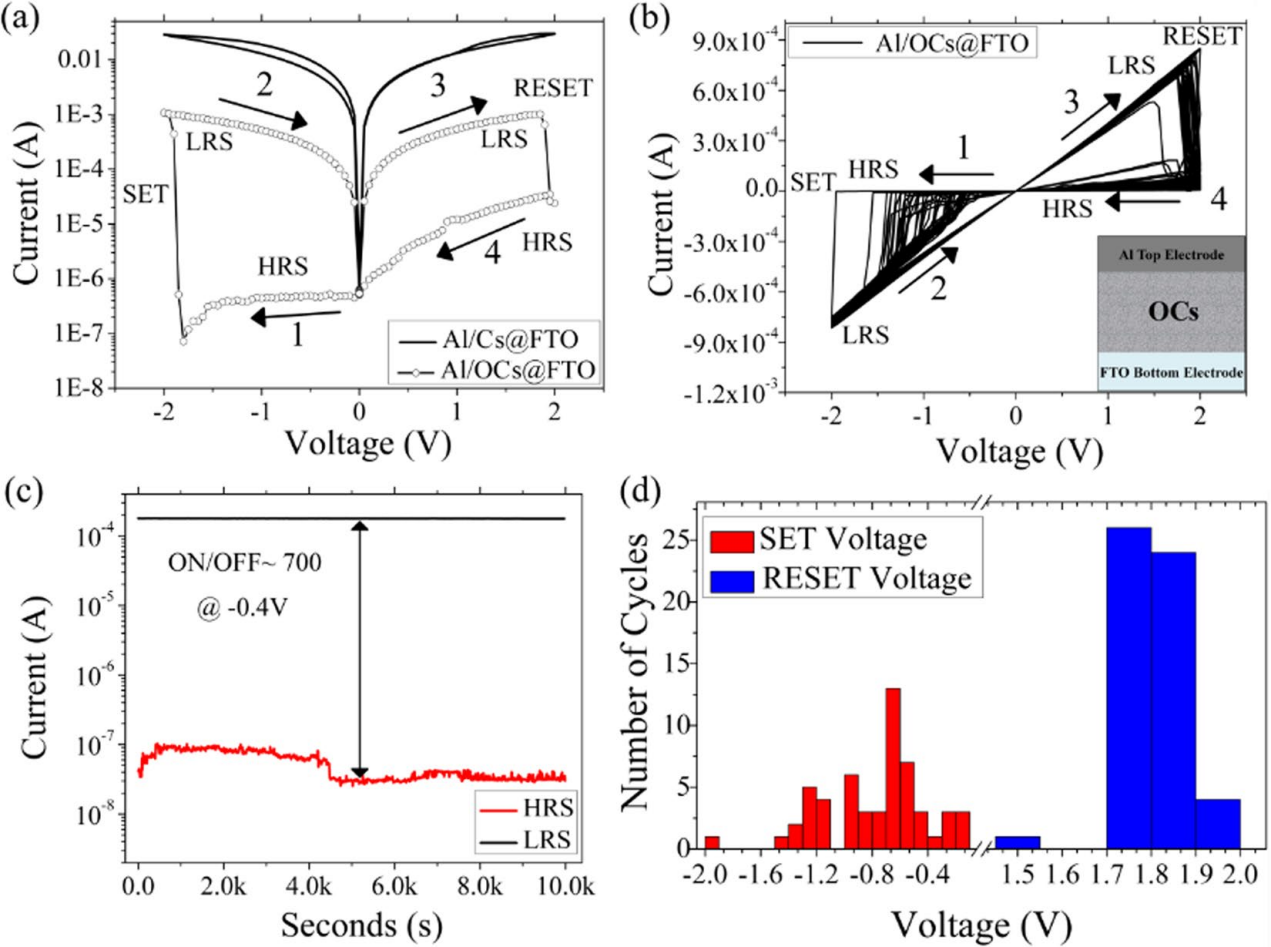
(**a**) I-V curve of Al/Cs@FTO and Al/OCs@FTO devices after 1 cycle of voltage sweeping. (**b**) Endurance of the Al/OCs@FTO device for over 50 cycles. (**c**) Retention results of the Al/OCs@FTO device over 10 × 10^3^ seconds at a reading voltage of −0.4 V. (**d**) Distribution of the SET and RESET voltages of the Al/OCs@FTO device.

To investigate the electrical performances of the samples, we fabricated Al/Cs@FTO and Al/OCs@FTO devices by depositing Al electrodes on top of the Cs and OCs, following the procedure described in Section 2.3. A schematic of the Al/OCs@FTO device is shown as inset in Fig. 3b. A sweeping voltage of 0 V → −2V → 0 V → 2 V → 0 V was applied in both devices and the results have been plotted in Fig. 3a. The I-V curve in Fig. 3a shows that Al/Cs@FTO and Al/OCs@FTO devices possess a bipolar resistive switching behavior. Noteworthy, the Al/Cs@FTO device is initially in the LRS due to presence of a higher percentage of sp^2^ carbon atoms, which provide high conductive channels^22^. The device is then switched to the HRS upon application of a voltage from −2 V to 0 V, which is maintained up to 2 V. At this voltage the RESET process occurs and the device is switched again to the LRS state. Conversely, the Al/OCs@FTO I-V curve shows that initially the device is in the HRS state, which is attributed to the presence of a higher percentage of sp^3^ carbon atoms due to the electrochemical oxidation^19^. As the negative voltage increases the devices are switched (SET) to the ON (LRS) state, which is maintained during the application of positive voltages. At 2 V the devices are switched (RESET) to the OFF (HRS) state.

Comparing the I-V curves of Al/Cs@FTO and Al/OCs@FTO devices, it is possible to notice that the ON/OFF ratio value of the Al/OCs@FTO device is ~10^2^ higher than the Al/Cs@FTO one, and it could sustain up to 50 sweeping cycles, as displayed in Fig. 3b. It was recorded as well that there was a change in the SET and RESET voltages (Fig. 3d) due to the rupture, in different locations, of the conductive filaments. This behavior could be attributed to the surface roughness of the OCs (Fig. 2g), which may lead to localized electric-field enhancements leading to an early breakdown^51–53^. A study of the effects of the electrodeposition parameters on the surface roughness^54^, i.e. type of current (direct or pulsed), pulse current amplitude, current on-time, and current off-time, could be performed to improve the devices’ reliability. The retention characteristics of the Al/OCs@FTO device at −0.4 V (Fig. 3c) showed that the HRS and LRS states can be retained up to 1 × 10^4^ seconds, thus confirming the non-volatile nature of the Al/OCs@FTO device. In order to compare the electrical performances of our devices with a device containing graphene oxide (GO), we fabricated an Al/GO@FTO device drop-casting a solution of GO (Graphene Supermarket) on a FTO substrate. The obtained I-V curves are displayed in Fig. S4a. Due to the presence of sp^3^ carbon atoms, the Al/GO@FTO device is initially in the HRS state, and it is switched to LRS at higher voltage (−5 V) than Al/OCs@FTO device. The Al/GO@FTO device is nonvolatile, indeed the LRS is maintained during the sweeping voltage −5 V → 0 → 5 V and it can sustain up to 10 sweeping cycles. The device showed a good retention up to 1 × 10^4^ seconds at a reading voltage of −0.4 V; however, the ON/OFF ratio is two order of magnitude lower than the Al/OCs@FTO, which is too low for its application in commercial RRAM devices. From these results and comparison, it is evident that the electrochemical oxidation of the Cs enhanced the electrical performances of the device. The analysis of the chemical composition of the GO@FTO sample was performed by XPS and the results are shown in Fig. S5c. Compared to the XPS of the OCs sample, it was found a higher percentage of carboxyl groups (~35%) and a lower content of sp^3^ carbon atoms (17% for GO and 39% in the OCs). Moreover, the presence of carboxyl groups was detected (Table S1). From these comparisons, it seems that the differences in the electrical performances between the Al/OCs@FTO and Al/GO@FTO devices could be attributed to their different chemical compositions, however more investigations are required.

### Resistive Switching Mechanism

**Figure 4:**
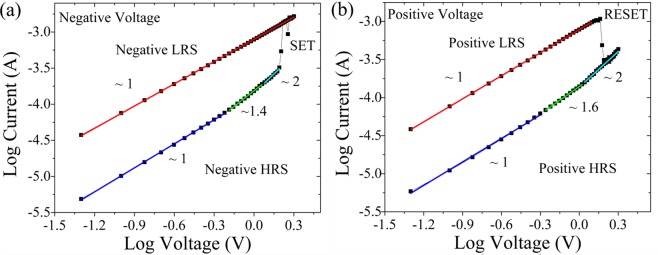
I-V curves of Al/OCs@FTO device plotted in a double logarithmic scale under negative (**a**) and positive (**b**) voltages.

From the XPS analysis it was found that upon anodic oxidation of Cs, a higher percentage of hydroxyl and epoxy groups was observed along with the appearance of the peak attributed to carbonyl groups (in Table S1 the surface composition of the Cs and OCS is provided). Furthermore, we have demonstrated that the presence of these oxygen functional groups, sp^3^ and sp^2^ carbon atoms play a key role in resistive switching behavior in RRAM devices^8–10,14,16–18,55^. It is important to understand the conduction mechanism in these type of devices to improve their performances. Fig. 4a,b shows the I-V curves of the LRS and HRS during SET and RESET operations fitted in a double logarithmic scale, respectively.

Under negative and positive voltages the LRS states follow the Ohm’s law conduction mechanism with a slope ~1 (red fitted lines in Fig. 4a,b). Instead, the negative and positive HRS states show a slope ~2 (cyan fitted lines) and are governed by the Child’s law conduction mechanism according to the relationship I (V) = αV + βV^2^. These results are in agreement with the SCLC mechanism and with the current literature regarding GO-based RRAM^4,18–20^. The resistive switching behavior in the OCs takes place through the formation of conductive filament paths built from the material’s oxygen vacancies and structural defects, which has been reported by several works^4,56–58^. The Al/OCs@FTO device can be then categorized as an oxygen vacancies based (VO) RRAM^16^, where the formation and rupture of oxygen vacancies conductive filaments is responsible for the resistive switching mechanism.

**Figure 5:**
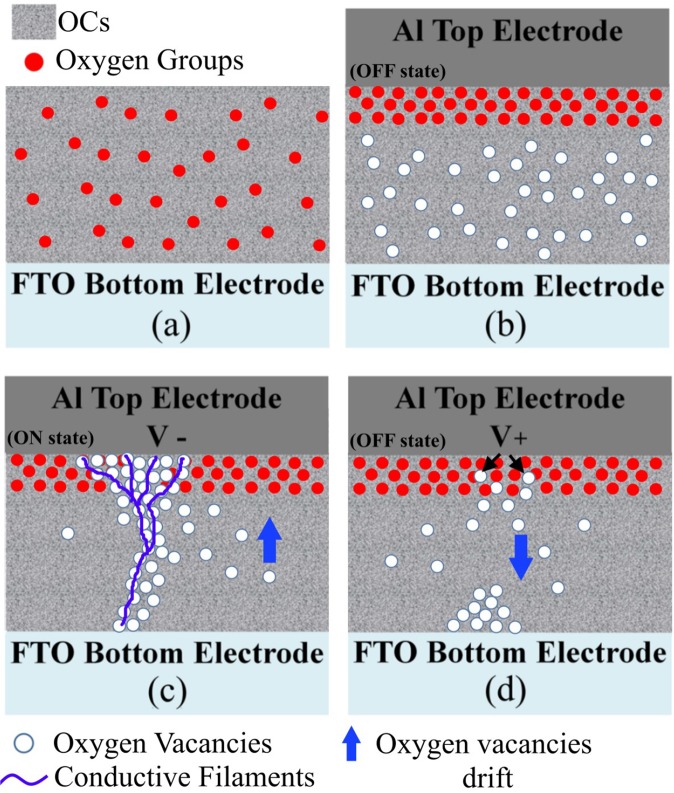
(**a**) OCs deposited onto the FTO electrode. (**b**) Pristine OCs device after deposition of the Al top electrode. (**c**) Drifting of the positively charged oxygen vacancies towards the bottom electrode upon application of a negative voltage and formation of the conductive filaments, which switch the device ON. (**d**) During the reset process the oxygen vacancies are repelled back from the Al top electrode causing the rupture of the conductive filaments and the switch of the device to the OFF state.

A proposed mechanism for the RS behavior of the Al/OCs@FTO device is outlined as a schematic in Fig. 5a,d. Pradhan and Kim^4,12^, studied the resistive switching mechanism in graphene oxide based RRAM devices and it was demonstrated that the RS behavior is attributed to the formation of conductive filaments. Aluminum has a high affinity to oxygen, therefore it reacts with the oxygen ions desorbed from the OCs matrix, which induces the formation of an oxygen-rich region near the top Al electrode.

This behavior has been demonstrated by the TEM-cross section analysis displayed in Figs S2 and S3. Indeed, from the composition maps it is possible to notice a higher percentage of oxygen near the top Al electrode. Because of the removal of oxygen from the OCs (due to the affinity with Al), oxygen-deficient regions (i.e. oxygen vacancies) are formed within the OCs matrix (Fig. 5b). We could not confirm the percentage of oxygen-containing groups remaining in the carbon structures due to difficulties in performing XPS analysis after the deposition of the Al top electrode, however this requires more attention. When a negative voltage is applied at the Al top electrode, the oxygen vacancies move towards the cathode and start clustering, inducing the formation of conductive filaments (Fig. 5c), which switch the device from the OFF to the ON state, i.e. SET process. During the RESET process, the positive bias pushes back the oxygen vacancies and the breakage of the conductive filaments occurs switching the device to the HRS state (Fig. 5d)^2,4^. The observation of different SET voltages with the number cycles (Fig. 3d), could be attributed to the fact that during the RESET process the conductive filaments can break at various locations leading to a distribution of SET voltages.

### Device Engineering

#### Electrophoretic Oxidation Engineering

In order to study how the degree of the Cs oxidation influences the electrical performances, we fabricated two devices with different electrochemical oxidation/reduction/oxidation cycles. In order to do so, the Cs were oxidized applying a voltage from 0 V to 0.8 V, then reduced applying a negative voltage from 0 V to −0.8 V. The reduction was then followed by another cycle of anodic oxidation in the range [0–0.8] V. The samples were obtained in a way, that the number of oxidation/reduction/oxidation cycles was 3 and 6, respectively. These samples will be referred as 3OCs and 6OCs, where the number indicates the number of anodic oxidation cycles performed. The XPS spectra of these devices are shown in Fig. S6a,b. In Table S1 the chemical compositions of the Cs are displayed before and after electrochemical treatment. Noteworthy, increasing the cycles of oxidation/reduction/oxidation the percentage of sp^2^ carbon atoms increases compared to the Cs oxidized with only one anodic oxidation. Furthermore, the percentage of epoxy groups is higher in the OCs compared to the 3OCs and 6OCs samples. The I-V curves of Al/3OCs@FTO and Al/6OCs@FTO devices are displayed in Figure S6(c), and it was observed that the electrical performances do not improve *via* increasing the number of oxidation/reduction/oxidation. The Al/3OCs@FTO electrical behavior is similar to the Al/Cs@FTO device; the device is initially in the LRS due to the higher percentage of sp^2^ carbon atoms and switch to HRS upon application of a voltage from −2 V to 0 V. However, the device cannot maintain the HRS state and switch to LRS upon application of a positive voltage. The I-V curves for the 6OCs showed that this device behaves like a resistor, due to the presence of a higher concentration of sp^2^ carbon atoms. These results highlight that the degree of oxidation of the Cs strongly influences their electrical performances, *i.e*. a higher concentration of sp^3^ carbon atoms along with the presence of carbonyl and epoxy groups improved the device’s performances. However, a higher percentage of carbonyl groups (C=O) does not reach high values of ON/OFF ratio in the devices. It is clear, that the amount of sp^3^, sp^2^ carbon atoms and oxygen containing carbon groups play a key role in electrical performances of the Al/OCs@FTO devices.

#### Salt Concentration Engineering

**Figure 6:**
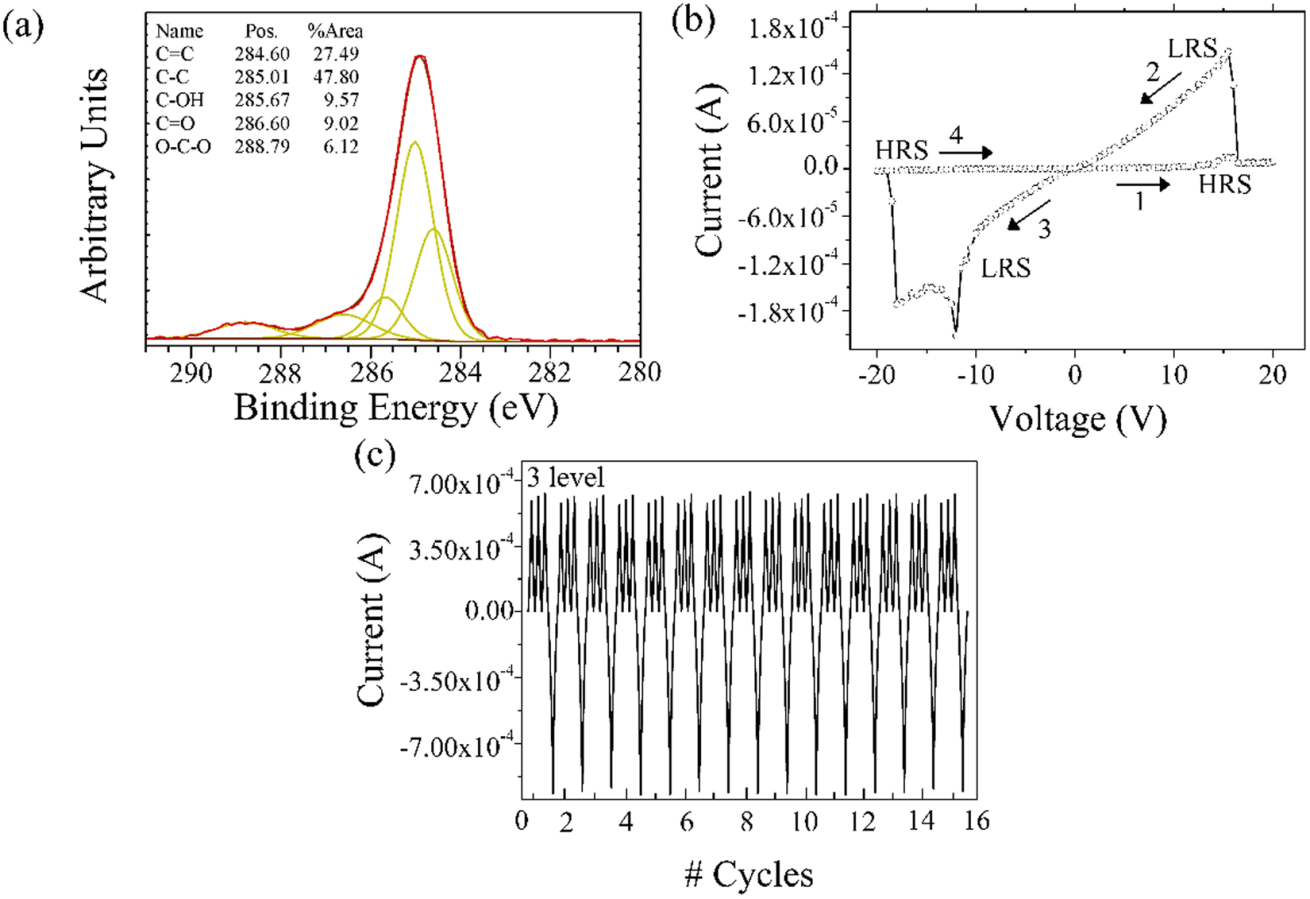
(**a**) XPS spectra of the 3MOCS, (**b**) current response under a voltage sweep of 20 V, (**c**) 3 level memory profile upon application of 15 V and a reset bias of −21 V.

The possibility to specifically control the degree of oxidation is necessary for the successful fabrication of next generation carbon-based RRAM devices. In order to extent the investigation on the role of oxygen content in our materials, we performed preliminary experiments carrying out the electrochemical oxidation increasing the salt concentration from 1 M to 3 M. The materials obtained under this anodic oxidation conditions, are indicated as 3MOCs. In Fig. 6a, is displayed the XPS spectrum of the 3MOCs and it was observed that the 3MOCs samples have a lower percentage of sp^2^ carbon atoms and hydroxyl groups and a higher percentage of carbonyl groups than the OCs sample (Table S1). The electrical measurements results, plotted in Fig. 6b, showed that compared to the Al/OCs@FTO device, a voltage of 15 V was required to switch the 3MOCs device from the OFF state to the ON state, due to the higher percentage of sp^3^ carbon atoms and carbonyl groups. Interestingly, the 3MOCs device showed a three-level memory effect upon the application of consecutive biasing of 15 V, as can be seen from Fig. 6c.

This phenomenon can be attributed to either the creation of multiple conduction paths under consecutive bias application, which is known to occur in oxide-based RRAM devices^27,59^ or the increase of the filaments’ diameter as reported by Gao and co-workers, which may lead to the creation of multiple atomic point contacts^60,61^. The application of a reverse erase bias of −21 V disrupts the conduction paths and the device goes back to its original state, allowing a repeatable 3 level current memory.

The results we presented in this work demonstrate that the electrochemical oxidation of carbon-materials could be used as an efficient tool to engineer their chemical composition. This in turns could lead to the fabrication of next generation RRAM devices with enhanced electrical performances, such as multilevel memory behavior for the storage of more than one bit per memory cell^62^.

## Conclusions

In summary, in this work we demonstrate that the electrochemical oxidation of carbon structures can be used as an environmentally friendly, time- and cost-effective technique to engineer the electrical performances of carbon-based memory devices for their use in next generation electronic applications. The preliminary results, demonstrated a good reproducibility of the Al/OCs@FTO devices, which show a bipolar resistive switching behavior under an operating voltage of 2 V and retention time of 10^4^ s. It was further demonstrated that the degree of oxidation plays a key role in the electrical properties of the devices. Indeed, it was observed that a proper control over the chemical composition of the Cs structure is vital for their resistive switching mechanism, since it is originated by the formation of conductive filament paths built from the oxygen vacancies and structural defects. Moreover, the increase of the Cs oxidation led to devices with multilevel memory which enables the storage of more than one bit of information. The simplicity and time effectiveness of the method we developed has the potential to be employed for the large-scale development of other carbon-based electronics.

## Supplementary information

Supporting Information
