# Supplementary material for: Electrochemical Oxidation Induced Multi-Level Memory in Carbon-Based Resistive Switching Devices
Source: Sci Rep. 2019 Feb 7;9:1564. doi: 10.1038/s41598-018-38249-0 (PMC6367418; doi:10.1038/s41598-018-38249-0)
Supplement: Supplementary file 1 — Supporting Information [file 41598_2018_38249_MOESM1_ESM.docx]

Electronic Supplementary Material

Electrochemical Oxidation Induced Multi-Level Memory in Carbon-Based Resistive Switching Devices

Paola Russo^1, 2, 3, 4^*, Ming Xiao^1, 2, 4^, Norman Y. Zhou^1, 2, 4^*

^1^Waterloo Institute for Nanotechnology, ^2^Centre for Advanced Materials Joining, ^3^Multi-Scale Additive Manufacturing Lab, ^4^Department of Mechanical and Mechatronics Engineering, University of Waterloo, 200 University Avenue West Waterloo, Ontario N2L 3G1, Canada

*Corresponding author: Centre for Advanced Materials Joining, Department of Mechanical and Mechatronics Engineering, University of Waterloo, 200 University Avenue West, Waterloo, Ontario N2L 3G1, Canada.

**E-mail addresses**: *[nzhou@uwaterloo.ca](mailto:nzhou@uwaterloo.ca) (Norman Y. Zhou).

KEYWORDS: electrochemical oxidation, carbon-based devices, X-ray photoelectron spectroscopy, device engineering, resistive switching, multilevel memory device

**Electronic Supplementary Material**

Electrochemical oxidation of FTO substrate…...……………….………...……………..….........…....S1

TEM-Cross section of the Al@Cs@FTO...............................................................................................S2

TEM-Cross section of the Al@OCs@FTO............................................................................................S3

Raman spectra of the as-prepared Cs and the OCs…………………………………………………….S4

Al/GO@FTO electrical performances and GO@FTO XPS spectrum…...............................................S5

XPS spectra and I-V curves of 3OCs and 6OCS devices.......................................................................S6

Table S1: Surface composition of the Cs before and after electrochemical treatment...........................S7

**Electrochemical oxidation of the FTO substrate**


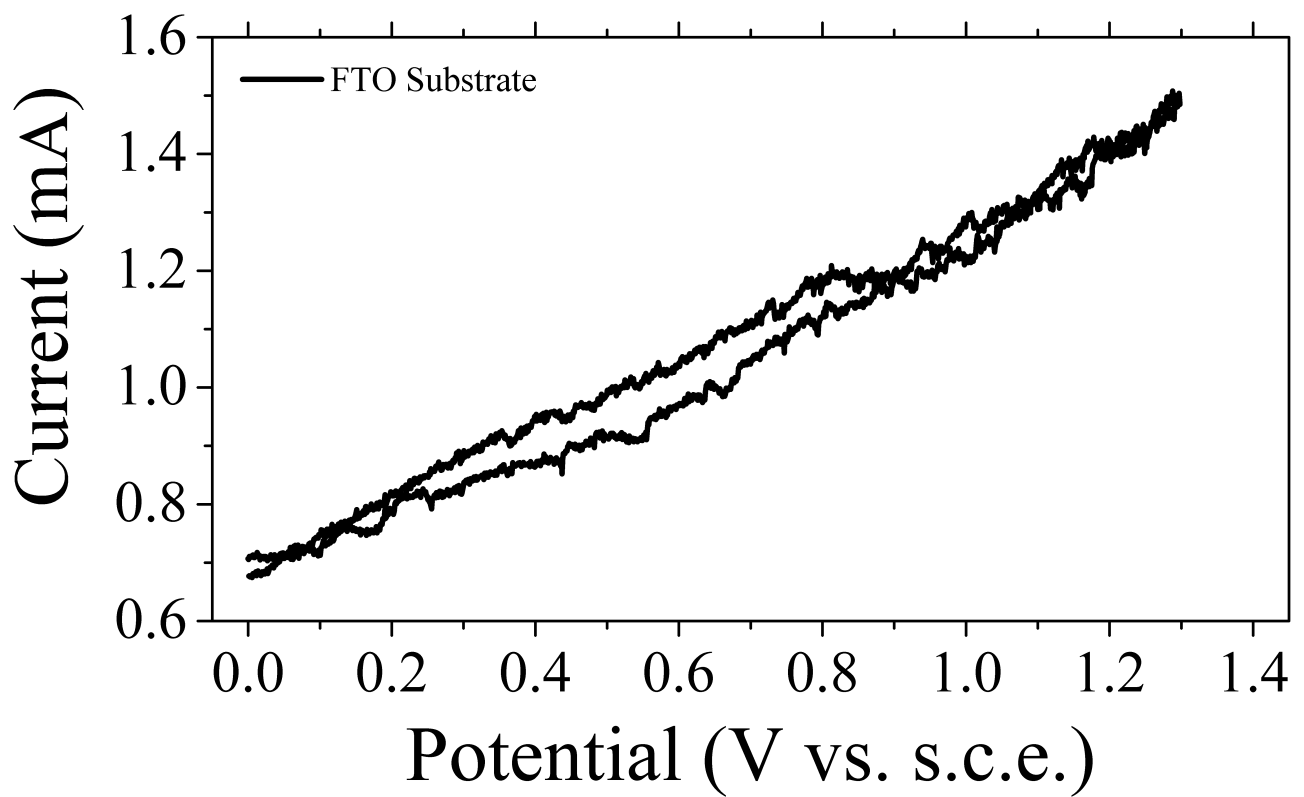


Figure S1: Electrochemical oxidation of the FTO substrate without the deposition of the carbon structures. It is possible to notice that the FTO surface has not been oxidized.

**TEM-Cross section of the Al@Cs@FTO**


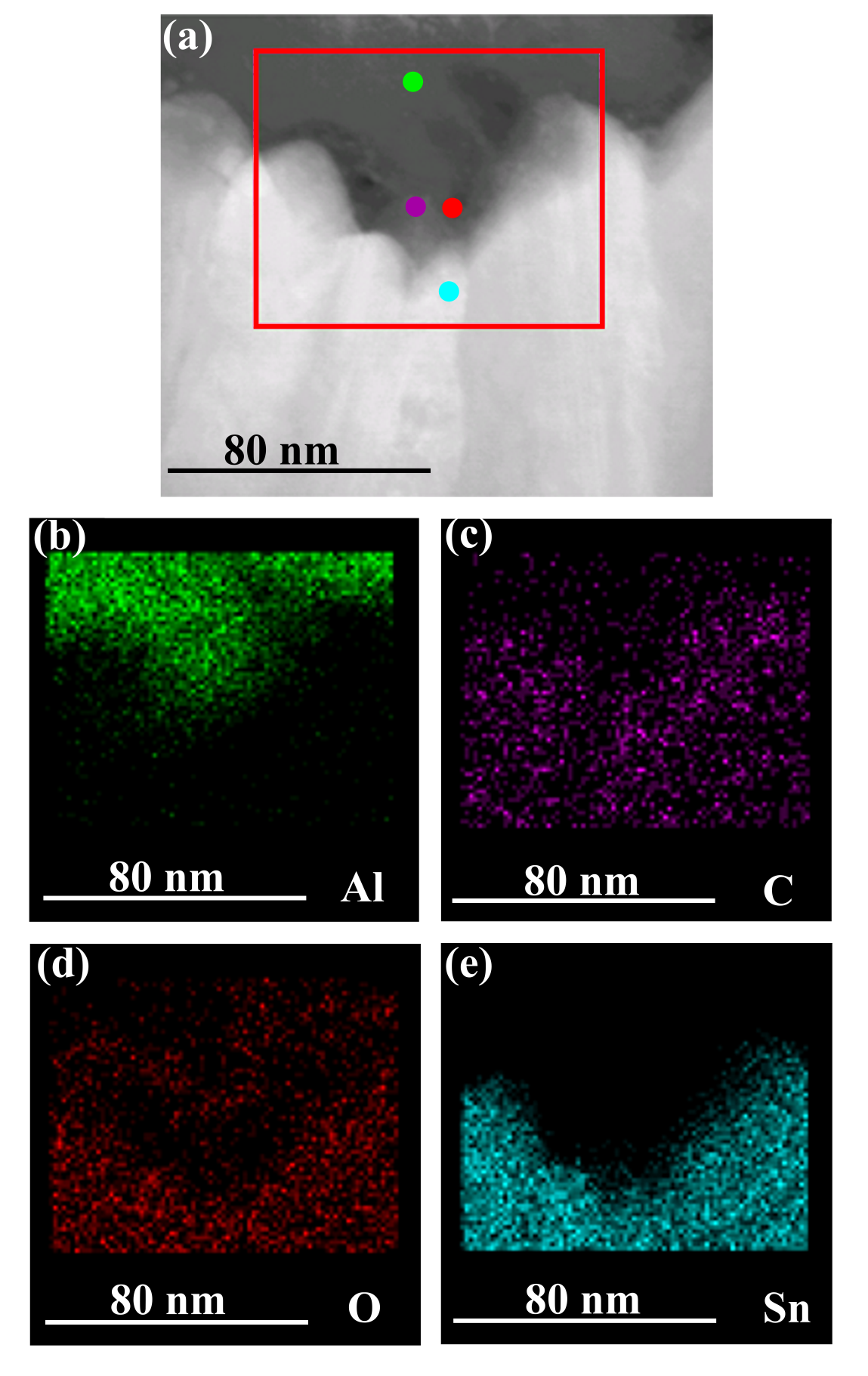


Figure S2: (a) TEM-Cross section of the Al@Cs@FTO device. (b-e) composition maps of Al, C, O and Sn, respectively.

**TEM-Cross section of the Al@OCs@FTO**


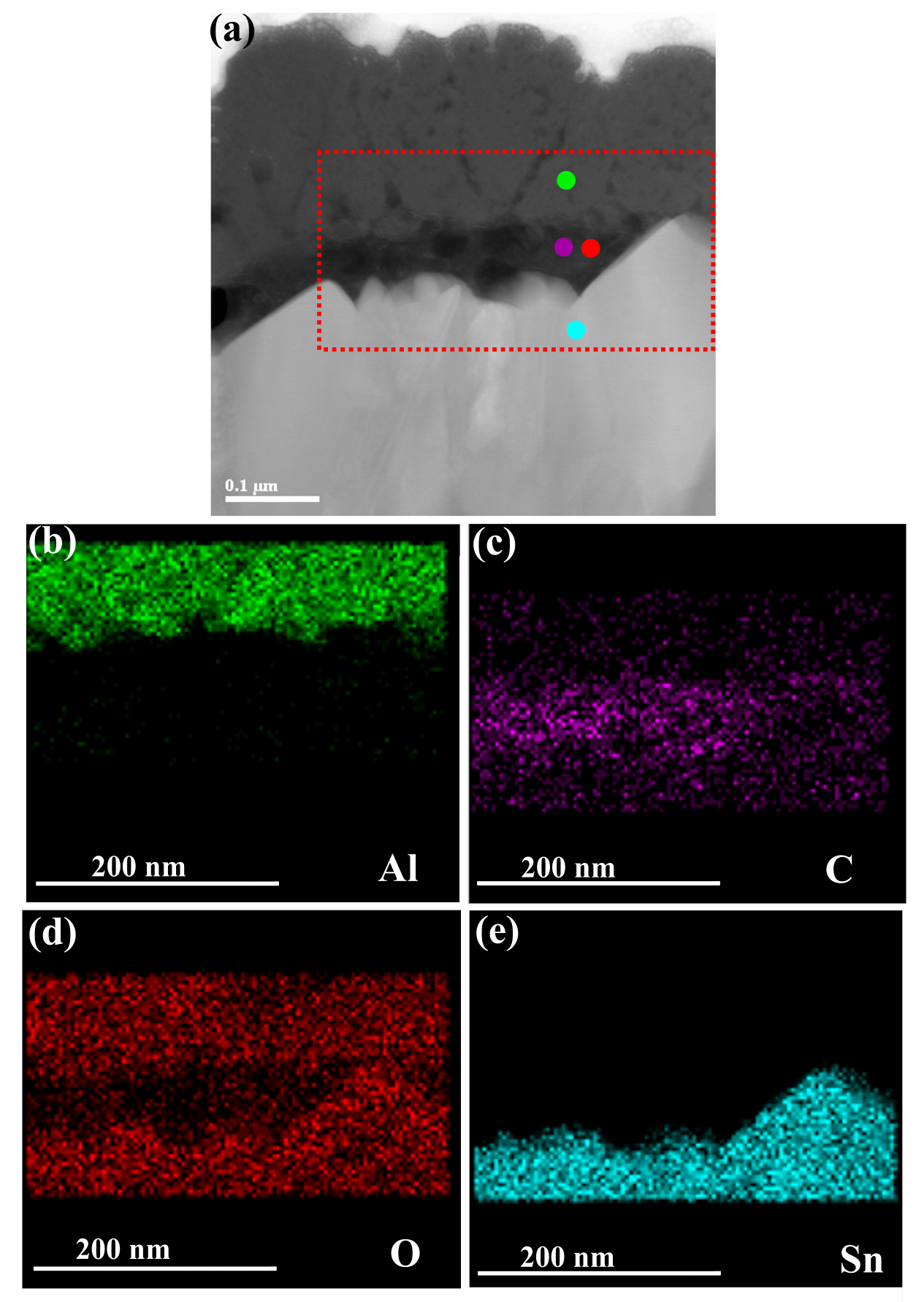


Figure S3: (a) TEM-Cross section of the Al@OCs@FTO device. (b-e) composition maps of Al, C, O and Sn, respectively.

**Raman spectra of the as-prepared Cs and the OCs**


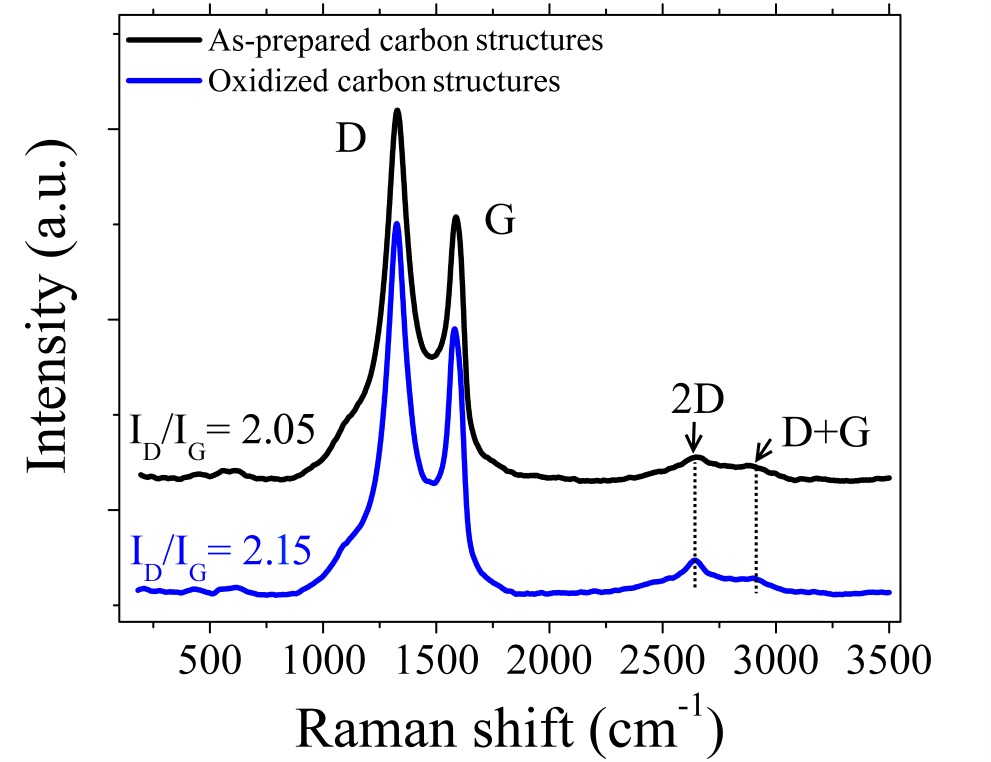


Figure S4: Raman spectra of as-prepared Cs (black curve) and the Cs after electrochemical oxidation (blue curve).

**Al/GO@FTO electrical performances and GO@FTO XPS spectrum**

**
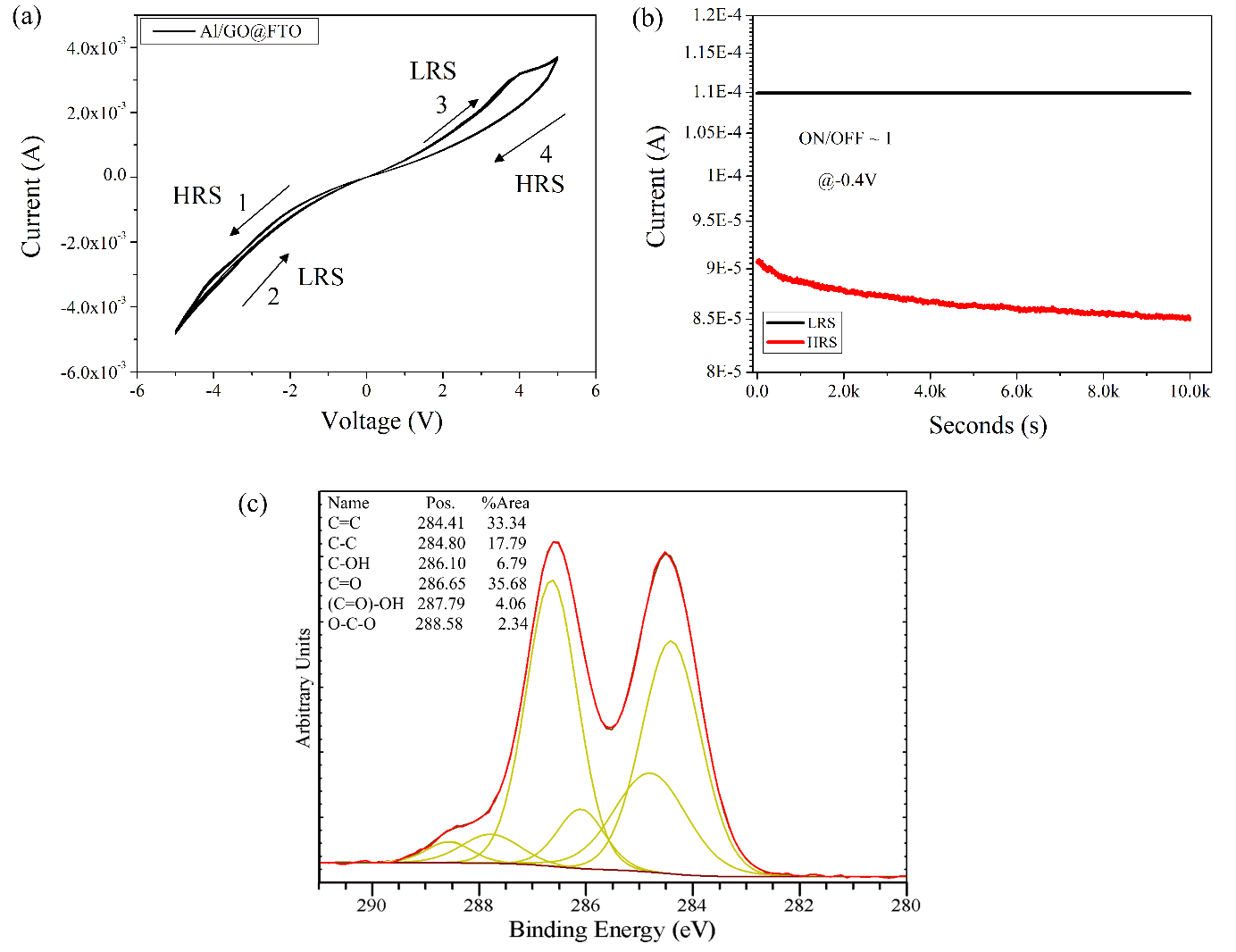
**

Figure S5: (a) I-V curves of Al/GO@FTO showing 10 cycles of voltage sweeping at 5V. (b) Retention results of the Al/OCs@FTO device over 1x10^4^ seconds at a reading voltage of -0.4V. (c) XPS of the GO sample.

**XPS spectra and I-V curves of 3OCs and 6OCS devices**

**
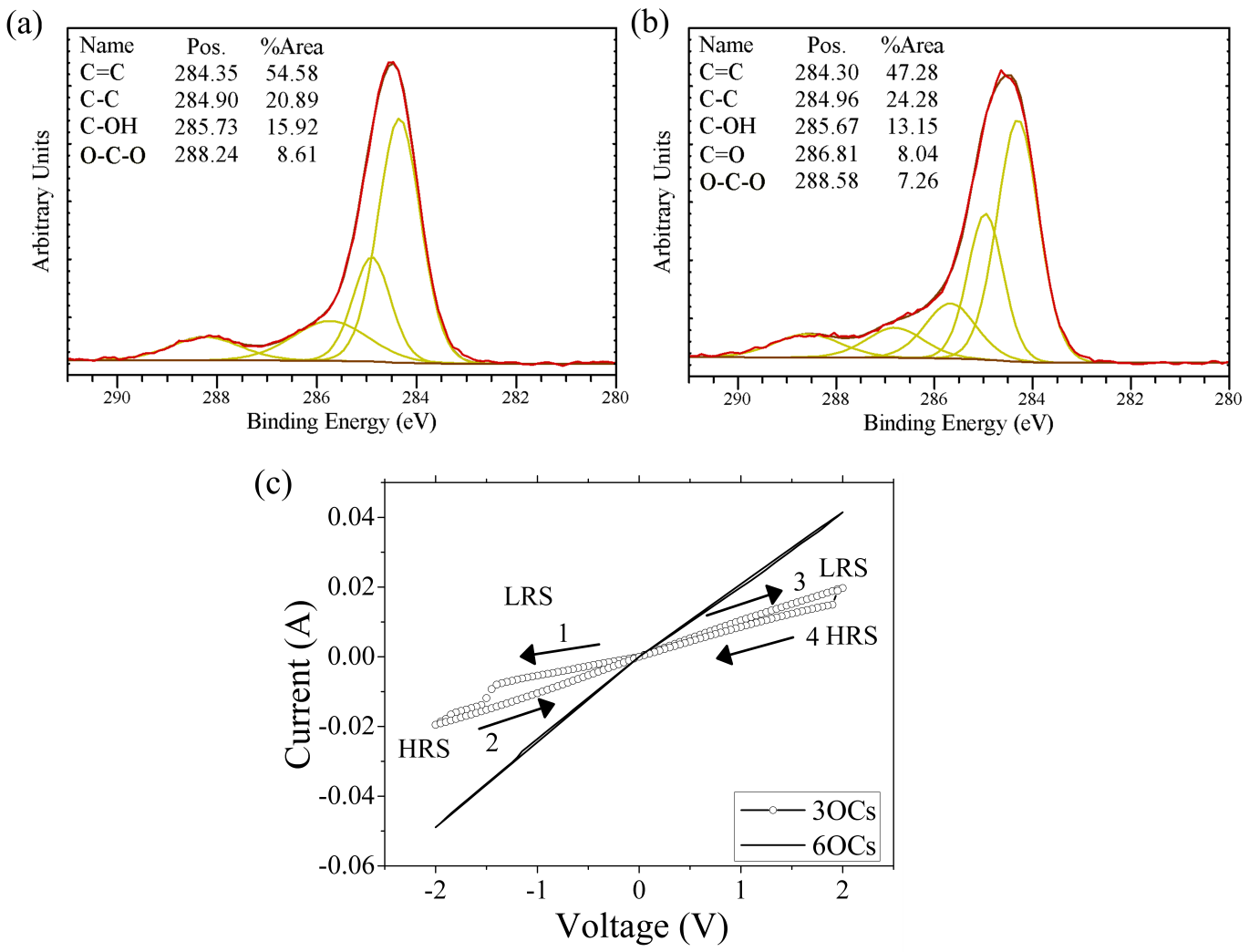
**

Figure S6: XPS spectrum of the Cs after 3 (a) and 6 (b) oxidation/reduction/oxidation cycles, respectively. (c) I-V curve of Al/3OCs@FTO and Al/6OCs@FTO devices after 1 cycle of voltage sweeping.

Table S1: Surface composition of the Cs before and after electrochemical treatment.

| Sample | C=C (%) | C-C (%) | C-OH (%) | C=O (%) | O-C-O (%) | (C=O)-C-OH (%) |
| --- | --- | --- | --- | --- | --- | --- |
| Cs | 70.25 | 14.70 | 9.97 | // | 5.08 | // |
| OCs | 30.80 | 39.66 | 14.41 | 3.59 | 11.54 | // |
| GO | 33.34 | 17.79 | 6.79 | 35.68 | 2.34 | 4.06 |
| 3OCs | 54.58 | 20.89 | 15.92 | // | 8.61 | // |
| 6OCs | 47.28 | 24.28 | 13.15 | 8.04 | 7.26 | // |
| 3MOCs | 27.49 | 47.80 | 9.57 | 9.02 | 6.12 | // |
